# The association between living habits, physical activity level and sarcopenia in chinese older adults with type 2 diabetes mellitus: a cross-sectional study

**DOI:** 10.3389/fpubh.2025.1608511

**Published:** 2025-09-03

**Authors:** Yiting Yue, Huaping Shen, Hongmei Li, Yongjing Zhang, Yiwei Zhao, Rui Zhang, Xiaoyan Xue

**Affiliations:** ^1^Department of Basic Nursing Teaching and Research, Fenyang College of Shanxi Medical University, Fenyang, China; ^2^Department of Medical Nursing Teaching and Research, Fenyang College of Shanxi Medical University, Fenyang, China; ^3^Department of Ultrasound, Fenyang Hospital, Fenyang, Shanxi, China; ^4^Department of Surgical Nursing Teaching and Research, Fenyang College of Shanxi Medical University, Fenyang, China

**Keywords:** older adults, type 2 diabetes mellitus, sarcopenia, lifestyle habits, physical activity

## Abstract

**Objective:**

Sarcopenia significantly impacts quality of life and increases morbidity in older adults with type 2 diabetes mellitus (T2DM). This study aimed to investigate the association between Living habits, physical activity levels, and sarcopenia in this population.

**Methods:**

A cross-sectional study included 65 older adults with T2DM from a tertiary hospital in Shanxi Province, China. Dietary habits were assessed using a 3-day dietary recall method. Physical activity levels were quantified using the International Physical Activity Questionnaire (IPAQ) and categorized (high/moderate/low) based on metabolic equivalent (MET)-minutes/week. Sleep patterns were evaluated using the Pittsburgh Sleep Quality Index (PSQI), with regular sleep defined as 7–9 h nightly and a PSQI score ≤ 5. Sarcopenia was diagnosed according to the 2019 Asian Working Group for Sarcopenia (AWGS) criteria. Participants were categorized into sarcopenia and non-sarcopenia groups. Clinical data were compared, and multivariate logistic regression analyzed associations between lifestyle factors, physical activity, and sarcopenia.

**Results:**

The proportions of regular exercise, regular sleep, balanced diet, and high physical activity were significantly lower in the sarcopenia group (*P* < 0.05). Multivariate analysis identified regular exercise (OR = 0.42, 95% CI: 0.25–0.71), regular sleep (OR = 0.56, 95% CI: 0.33–0.94), balanced diet (OR = 0.61, 95% CI: 0.37–0.99), and high physical activity (OR = 0.48, 95% CI: 0.29–0.80) as protective factors against sarcopenia (all *P* < 0.05).

**Conclusion:**

Living habits and physical activity levels are significantly associated with sarcopenia risk in older adults with T2DM, suggesting potential targets for prevention strategies.

## 1 Introduction

Diabetes is a common chronic disease in clinic, which often occurs in middle-aged and older adults people. Diabetes mellitus is a chronic metabolic disorder resulting from the interaction of environmental, genetic and other factors, characterized primarily by hyperglycemia. The disease is clinically classified into type 1 diabetes and type 2 diabetes mellitus (T2DM), with distinct pathophysiological mechanisms. Type 2 diabetes mellitus accounts for ~90–95% of all diabetes cases worldwide, making it the predominant form of the disease (1). China has gradually entered an aging society, and the prevalence rate of diabetes in the older adults exceeds 20%, which is one of the main factors endangering the health of the older adults (2).

Sarcopenia is one of the common complications in older adults patients with T2DM. Epidemiological studies show that the prevalence of sarcopenia in older adults with T2DM ranges from ~15 to 40%, significantly higher than in age-matched individuals without diabetes (3). Sarcopenia is characterized by progressive loss of skeletal muscle mass and function, leading to reduced physical performance and increased risk of falls and fractures in patients (4). Previous clinical studies have considered (5) that sarcopenia is closely related to human metabolism, especially with diabetic patients. The two promote each other and form a vicious circle, which not only significantly increases the risk of falls and fractures, but also is closely related to cardiovascular events, decreased quality of life and increased mortality. In addition, some studies have shown (6) that sarcopenia is an important complication of diabetic patients and one of the signs of systemic function decline. The prevention and diagnosis of sarcopenia in older adults diabetic patients is helpful to delay the progression of weakness in older adults patients.

The development and maintenance of the musculoskeletal system is significantly influenced by lifestyle factors, particularly physical activity patterns (7). Regular exercise, especially resistance training, stimulates muscle protein synthesis, enhances muscle strength, and may counteract age-related muscle loss, by up-regulating the mTOR–p70S6K pathway, stimulates muscle protein synthesis, and attenuates ubiquitin–proteasome-mediated proteolysis (8, 9). Adequate dietary protein (≥ 0.8 g · kg^−1^ · day^−1^) evenly distributed across meals maximizes muscle protein anabolism and provides essential amino acids for reconditioning (10). Beyond exercise and diet, sleep duration and quality influence muscle recovery through nocturnal growth-hormone and testosterone secretion; short or fragmented sleep elevates cortisol, impairs glucose tolerance, and accelerates muscle catabolism (11). Sleep disturbances can induce insulin resistance, elevate inflammatory cytokines (IL-6, TNF-α et al.), and impair muscle recovery (12). Despite established knowledge on individual lifestyle factors (13), the specific interplay between the composite construct of “living habits” [explicitly defined here as the combination of exercise habits, dietary patterns, and sleep behaviors (14)] and physical activity levels with sarcopenia risk in older Chinese adults with T2DM remains incompletely characterized.

In view of this, the present study aims to analyse the relationship between living habits and physical activity and sarcopenia in older adults patients with T2DM. We hypothesize that: (1) Regular exercise habits, a balanced diet, and consistent sleep patterns will be inversely associated with sarcopenia risk; (2) Higher overall physical activity levels [particularly high metabolic equivalent of task (MET) values] will be protective against sarcopenia; and (3) Patients exhibiting a combination of positive lifestyle habits will demonstrate the lowest prevalence of sarcopenia. The findings aim to provide a more comprehensive evidence base for targeted, multifaceted lifestyle interventions in clinical sarcopenia prevention strategies for this vulnerable population.

## 2 Materials and methods

### 2.1 Research participants

A total of 65 old patients with T2DM admitted to a tertiary hospital in Shanxi Province from March 2023 to March 2024 were selected as the research participants. Inclusion criteria: (1) meeting the diagnostic criteria for T2DM (15); (2) Age ≥ 60 years old, which is stipulated by the 2020 Chinese guidelines for prevention and treatment of T2DM in older adults (15) and widely adopted in sarcopenia research across Asia (16); (3) The patient's cognitive function was assessed using the Montreal Cognitive Assessment (MoCA), with scores ≥26 considered normal; (4) The course of T2DM is ≥ 5 years, as verified through medical records. Exclusion criteria: (1) Patients with severe organic diseases (defined as severe heart, liver, kidney failure, or advanced cancer); (2) Patients with mental disorders, depression and other diseases; (3) Disabled persons (defined as bedridden or unable to complete physical assessment independently); (4) Past history of bariatric surgery; (5) Patients with stents or pacemakers.

This study was approved by the Ethics Committee of the tertiary hospital in Shanxi Province (approval number: 2,024,068, date: December 17, 2024). Informed consent signed by the participant (or legal proxy for those with limited literacy).

### 2.2 General data collection

The average length of hospital stay was 9.24 ± 2.13 days. Data were collected during the fifth calendar day of hospitalization, once medical stability was confirmed. A single trained research dietitian and a geriatric nurse, both blinded to sarcopenia status, conducted all assessments in a quiet consultation room. Collect general data of patients, including age, gender, body mass index (BMI), marital status, education level, family income level, living habits (including eating habits, exercise habits, sleep habits, smoking and drinking habits), past medical history (hypertension, coronary heart disease) and diabetes-related complications (diabetic nephropathy, diabetic peripheral neuropathy), all obtained from medical records rather than self-report, fasting blood glucose, etc.

Dietary intake was captured with a validated 3-day dietary recall (2 weekdays + 1 weekend day) following Chinese Dietary Guidelines protocol (17). Portion sizes were quantified with calibrated digital kitchen scales (Tanita KD-400, Tokyo, Japan) and standard household measures, then analyzed with Nutritionist IV software (version 3.5, First DataBank, San Bruno, CA, USA) using the China Food Composition Database 2020. A “balanced diet” was defined as meeting the Chinese Nutrition Society recommendations for older adults: regular meals, diverse foods, daily protein 1.0–1.2 g kg^−1^ body weight, carbohydrates 50–60% of total energy, protein 10–15%, and fat 20–30% (Dietary Guidelines for Chinese 2022) (18).

Sleep behavior was documented with the Chinese version of the Pittsburgh Sleep Quality Index (PSQI; Cronbach's α = 0.82) (19); “regular sleep” was operationalised as 7–9 h night-time sleep according to National Sleep Foundation standards (20) with a PSQI global score ≤ 5 (21). Smoking status was ascertained with the WHO STEPS instrument (https://www.who.int/teams/noncommunicable-diseases/surveillance/systems-tools/steps/instrument) and categorized as never, former (> 12 months abstinence), or current. Alcohol consumption was quantified by the AUDIT-C; “current drinking” was defined as any alcohol use within the past 12 months (22). Cognitive function was screened with the Montreal Cognitive Assessment (MoCA) validated in Mandarin with a score ≥ 26 was considered normal (23).

### 2.3 Physical activity level

Physical activity was quantified using the validated Chinese version of the International Physical Activity Questionnaire (IPAQ) (24), administered through face-to-face interviews by trained staff. Exercise ≥ 3 times a week, each time ≥ 30 min is regular exercise. Data processing followed WHO protocols, with metabolic equivalent (MET)-minutes/week calculated according to the frequency, time and intensity of various physical activities performed by the participants in the past 7 days (25).

Activity levels were classified per WHO 2020 guidelines (26): high level: any strenuous physical activity for more than 3 days with total MET ≥ 1,500, or various combinations of physical activity within 7 days with total MET ≥ 3,000; Moderate level: at least 20 min of vigorous physical activity per day for more than 3 days, or at least 30 min of moderate-intensity physical activity or walking per day for more than 5 days, or various combinations of physical activity for more than 5 days with a total MET ≥ 600; Low level: those who do not meet the medium level standard.

### 2.4 Criteria for sarcopenia

Referring to the 2019 Asian Sarcopenia Working Group (AWGS) standard (27), all patients were detected by bioelectrical impedance method, and the data of skeletal muscle and body fat of patients were obtained by Korean Biospace Inbody 720 body composition tester, and the limb skeletal muscle index was calculated at the same time. The formula was limb skeletal muscle mass/height^2^, and skeletal muscle parameters = skeletal muscle mass/weight. The electronic grip force meter was used to measure the grip strength of both hands of the patient three times, and the maximum value was taken. According to the skeletal muscle index of limbs, the index ≤ 7.0 kg/m^2^ (male), ≤ 5.7 kg/m^2^ (female) or the maximum grip strength < 28 kg (male) and < 18 kg (female) as the standard, it can be judged as sarcopenia according to AWGS 2019 criteria (27). According to sarcopenia criteria, they were divided into sarcopenia group and non-sarcopenia group.

### 2.5 Sample size calculation

Sample size was calculated using G^*^Power 3.1 software. Based on previous studies on the association between lifestyle habits and sarcopenia (OR = 0.35–0.60), with two-sided test, significance level α = 0.05, and statistical power (1-β) = 0.80, the minimum sample size required was 60 cases. Considering a potential 10% data loss, we determined to include no fewer than 65 participants.

### 2.6 Statistical methods

All data were analyzed using the statistical software SPSS 25.0. Categorical data were expressed as frequencies and percentages, and compared using the χ^2^ test; continuous data were expressed as mean ± standard deviation (± s), and compared using the *t*-test.

To preliminarily explore the direction and strength of associations between key lifestyle factors and sarcopenia status prior to regression modeling, bivariate correlation analyses were performed. Given the binary nature of the sarcopenia outcome variable (0 = non-sarcopenia, 1 = sarcopenia) and the ordinal/categorical nature of the lifestyle factors (often coded dichotomously), point-biserial correlations were computed (28). Univariate logistic regression analyses were initially conducted to identify potential risk factors associated with sarcopenia; variables yielding a significance level of *P* < 0.1 in univariate analysis were subsequently entered into the multivariate logistic regression model. Variables known to be clinically relevant confounders (age, gender, BMI, and disease duration) were also included in the multivariate model regardless of their univariate significance. In the multivariate regression model, sarcopenia status was the dependent variable (coded as 0 = non-sarcopenia, 1 = sarcopenia). Independent variables included lifestyle habits (exercise: 0 = regular, 1 = irregular; sleep: 0 = regular, 1 = irregular; diet: 0 = balanced, 1 = unbalanced) and physical activity level (0 = high, 1 = moderate/low), while controlling for age (continuous), gender (0 = female, 1 = male), BMI (continuous), and disease duration (continuous). The Hosmer-Lemeshow test was used to assess goodness-of-fit of the model (χ^2^ = 6.352, *P* = 0.608), indicating adequate model fit.

Results are presented as odds ratios (OR) with 95% confidence intervals (95% CI). A *P* < 0.05 was considered statistically significant.

## 3 Results

### 3.1 Comparison of general data between the two groups of patients

**Figure 1:**
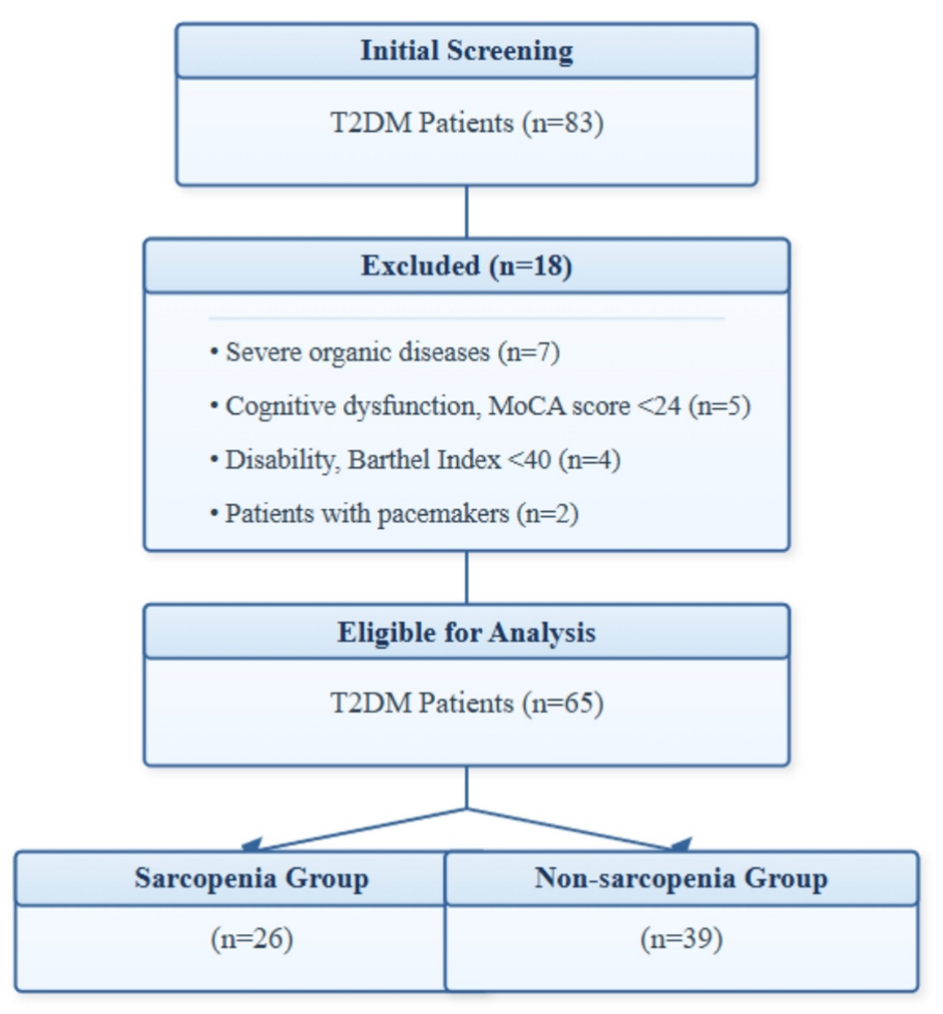
Patient flow diagram.

Figure 1 shows the participant flow chart of this study. Initially, 83 patients were screened, of which 18 were excluded (7 with severe organic disease, 5 with cognitive abnormalities, 4 with disabilities, and 2 with pacemakers). Upon diagnosis, out of the remaining 65 patients, 26 patients with sarcopenia were included in the sarcopenia group, and 39 patients without sarcopenia were included in the non-sarcopenia group. There was no statistically significant difference in the basic data between the two groups of patients (*P* > 0.05), see Table 1.

**Table 1:** Comparison of general data between the two groups of patients [cases (%), ± s].

**Information**	**Sarcopenia group (*n* = 26)**	**Non-sarcopenia group (*n* = 39)**	**χ^2^/t Value**	***P*-value**
**Gender (cases)**			1.241	0.265
Male	15 (57.69)	17 (43.59)		
Female	11 (42.31)	22 (56.41)		
Age (years)	67.82 ± 4.35	68.20 ± 5.07	0.313	0.755
BMI (kg/m^2^)	24.58 ± 2.16	24.89 ± 2.03	0.588	0.559
Course of disease (years)	6.14 ± 1.53	6.37 ± 1.66	0.564	0.575
**Marital status (cases)**			0.742	0.389
Married	21 (80.77)	34 (87.18)		
Other	5 (19.23)	5 (12.82)		
**Level of education (cases)**			1.356	0.244
Junior high school and below	15 (57.69)	17 (43.59)		
High school and above	11 (42.31)	22 (56.41)		
**Monthly income (yuan)**			0.864	0.353
< 3,000	14 (53.85)	17 (43.59)		
≥3,000	12 (46.15)	22 (56.41)		
**Past history (cases)**				
Hypertension	10 (38.46)	19 (48.72)	0.664	0.415
Coronary heart disease	8 (30.77)	9 (23.08)	0.475	0.491
**Complications (cases)**				
Diabetic nephropathy	7 (26.92)	8 (20.51)	0.364	0.546
Peripheral neuropathy	9 (34.62)	11 (28.21)	0.298	0.585
Fasting blood glucose (mmol/L)	11.59 ± 2.48	11.16 ± 2.53	0.677	0.501

### 3.2 Comparison of living habits and physical activity levels between the two groups of patients

The proportion of regular exercise, regular sleep, balanced diet and high physical activity level in the sarcopenia group were lower than that in the non-sarcopenia group (*P* < 0.05), see Table 2.

**Table 2:** Comparison of living habits and physical activity levels between the two groups (cases, %).

**Information**		**Sarcopenia group (*n* = 26)**	**Non-sarcopenia group (*n* = 39)**	**χ^2^ value**	***P*-value**
Living habits	Exercise habits (cases)			5.171	0.023
	Regular exercise	6 (23.08)	20 (51.28)		
	Occasional or inactive exercise	20 (76.92)	19 (48.72)		
	Sleep habits (cases)			6.448	0.011
	Regularity	9 (34.62)	26 (66.67)		
	Irregularity	17 (65.38)	13 (33.33)		
	Smoking (cases)			0.684	0.408
	Yes	12 (46.15)	14 (35.90)		
	No	14 (53.85)	25 (64.10)		
	Alcohol consumption (cases)			1.754	0.185
	Yes	17 (65.38)	19 (48.72)		
	No	9 (34.62)	20 (51.28)		
	Diet (cases)			4.524	0.033
	Equilibrium	9 (34.62)	24(61.54)		
	Disequilibrium	17 (65.38)	15(38.46)		
**Physical activity level (cases)**				5.489	0.019
High		7(26.92)	22 (56.41)		
Medium low		19(73.08)	17 (43.59)		

### 3.3 Multivariate logistic regression analysis of living habits, physical activity level and sarcopenia in older adults patients with T2DM

Taking older adults patients with T2DM sarcopenia as dependent variable (0 = non-sarcopenia, 1 = sarcopenia), living habits (exercise habits, sleep habits, food intake) and physical activity level as independent variables, and including age, gender, BMI, disease course and other possible confounding factors for multivariate Logistic regression analysis. Correlation analysis was performed to examine relationships between key variables, with regular exercise (*r* = −0.282, *P* = 0.023), regular sleep (*r* = −0.315, *P* = 0.011), balanced diet (*r* = −0.264, *P* = 0.034), and high physical activity level (*r* = −0.291, *P* = 0.019) showing significant negative correlations with sarcopenia (Table 3).

**Table 3:** Point-biserial correlations between lifestyle factors and sarcopenia status.

**Lifestyle factor**	**Correlation coefficient (*r*)**	***P*-value**
Regular exercise	−0.282	0.023
Regular sleep	−0.315	0.011
Balanced diet	−0.264	0.034
High physical activity	−0.291	0.019

Subsequent univariate logistic regression identified these factors as significantly associated with lower sarcopenia risk: regular exercise (OR = 0.42, 95% CI: 0.25–0.71, *P* = 0.001), regular sleep patterns (OR = 0.56, 95% CI: 0.33–0.94, *P* = 0.028), balanced diet (OR = 0.61, 95% CI: 0.37–0.99, *P* = 0.045), and high physical activity levels (OR = 0.48, 95% CI: 0.29–0.80, *P* = 0.004) were associated with lower risk of sarcopenia. Univariate logistic regression analysis identified regular exercise (OR = 0.29, 95% CI: 0.10–0.86, *P* = 0.026), regular sleep patterns (OR = 0.26, 95% CI: 0.09–0.78, *P* = 0.016), balanced diet (OR = 0.33, 95% CI: 0.11–0.96, *P* = 0.041), and high physical activity levels (OR = 0.28, 95% CI: 0.10–0.84, *P* = 0.023) as factors significantly associated with lower risk of sarcopenia. See Tables 4, 5.

**Table 4:** Multivariate logistic regression analysis of living habits, physical activity level and sarcopenia in older adults patients with T2DM.

**Factor**	**β value**	**SE**	**Wald χ^2^ value**	**OR value (95% CI)**	***P*-value**
Regular exercise	−0.868	0.269	10.424	0.42 (0.25–0.71)	0.001
Regular sleep	−0.579	0.263	4.845	0.56 (0.33–0.94)	0.028
Balanced diet	−0.494	0.247	4.002	0.61 (0.37–0.99)	0.045
High level of physical activity	−0.734	0.258	8.103	0.48 (0.29–0.80)	0.004

**Table 5:** Results of the univariate logistic regression analysis.

**Factor**	**OR value**	**95% CI**	***P*-value**
Regular exercise	0.29	0.10–0.86	0.026
Regular sleep	0.26	0.09–0.78	0.016
Balanced diet	0.33	0.11–0.96	0.041
High level of physical activity	0.28	0.10–0.84	0.023
Gender (male)	1.77	0.65–4.82	0.266
Age	0.98	0.89–1.08	0.753
BMI	0.93	0.74–1.18	0.557
Course of disease	0.91	0.67–1.24	0.574
Smoking (cases)	1.53	0.56–4.14	0.409
Alcohol consumption (cases)	1.99	0.72–5.54	0.188

## 4 Discussion

This cross-sectional study aimed to investigate the association between integrated lifestyle habits—encompassing exercise, dietary patterns, and sleep behaviors—and physical activity levels with sarcopenia risk in Chinese older adults with T2DM. Our key findings demonstrate that regular exercise habits, balanced nutrition, consistent sleep patterns, and higher physical activity levels are significantly associated with reduced sarcopenia prevalence. Multivariate analysis revealed these factors conferred substantial protection, with regular exercise exhibiting the strongest effect (OR = 0.42). These results indicate that older adults T2DM patients with sarcopenia have worse exercise, sleep, diet, and physical activity levels than patients with T2DM alone. This conclusion is basically consistent with previous studies. Lin et al. (29) showed that regular exercise can reduce the risk of sarcopenia in patients with T2DM. Pana et al. (30) found that good sleep quality was negatively correlated with the risk of sarcopenia.

From the mechanism analysis, firstly, the mass, strength and function of human skeletal muscle change constantly during human growth and development, and the state of skeletal muscle remains unchanged or decreases slightly in middle age, and then the muscle mass gradually decreases with age (31). Studies have shown (32) that the pathogenesis of sarcopenia is related to endocrine and metabolic disorders, chronic inflammation and other factors. Exercise has certain anti-inflammatory function, which can affect the level of a series of inflammatory factors, such as inhibiting the expression of tumor necrosis factor α in skeletal muscle, and then exert the effect of promoting protein synthesis, resisting muscle decomposition and enhancing muscle strength (33).

Secondly, patients with less exercise have a relatively higher proportion of connective tissue such as fat in their muscles, and their muscle contractility is significantly reduced (34); In addition, exercise can also stimulate the secretion of irisin, which is involved in the synthesis of brain-derived neurotrophic factor, which can promote skeletal muscle hypertrophy and repair muscle atrophy caused by denervation through autocrine, and achieve a protective effect on skeletal muscle (35). Thirdly, lack of sleep or sleep disorders can cause stress reaction in patients, activate the hypothalamus-pituitary-adrenaline axis, and then excite sympathetic nerves, cause insulin resistance and reduce insulin sensitivity, and then reduce insulin levels, ultimately leading to decreased muscle protein synthesis, leading to the risk of sarcopenia (36).

Finally, food intake is also associated with sarcopenia, and whether too much or too little food intake can affect the skeletal muscle of patients; Less intake of protein, unsaturated fatty acids and vitamin D deficiency not only affect the sleep quality of patients, but also lead to the decrease of muscle synthesis, resulting in the risk of sarcopenia (37–39).

Our findings align with recent systematic reviews and meta-analyses that consistently demonstrate the protective effects of regular physical activity against sarcopenia in older adults with T2DM. A Bayesian network meta-analysis showing that combinations of resistance training prescription could improve muscle strength and hypertrophy (40). Similarly, Izquierdo et al. emphasized that the protective effects of exercise on muscle health operate through multiple pathways, including improved insulin sensitivity, reduced inflammation, and enhanced myokine production (41). While our cross-sectional study cannot establish causality, the observed associations are consistent with these proposed mechanisms and highlight the potential for lifestyle interventions in clinical practice. The clinical implications of our findings are significant for the development of sarcopenia prevention strategies in older adults with T2DM. Clinicians should routinely assess lifestyle habits and physical activity levels during regular diabetes follow-up visits. This could involve implementing standardized screening tools for sarcopenia risk that incorporate lifestyle factors as predictors. Furthermore, individualized exercise prescriptions that consider the patient's physical capabilities and preferences would likely yield better adherence. The relationship between sleep quality and sarcopenia revealed in our study suggests that sleep health education should be integrated into diabetes management programs. Additionally, nutritional counseling focusing on adequate protein intake (0.8–1.2 g/kg/day) and optimal distribution throughout the day may help counteract the accelerated muscle loss in this population. A multidisciplinary approach involving endocrinologists, physical therapists, exercise physiologists, nutritionists, and sleep specialists would be ideal for comprehensive intervention.

Based on the results and mechanism of action of this study, the following preventive measures should be taken: (1) Encourage patients to engage in regular exercise, with an emphasis on progressive resistance training combined with moderate-intensity aerobic exercises. While low-intensity activities such as walking and Tai Chi may be appropriate starting points for deconditioned individuals, progression to resistance training is important for optimal muscle health as recommended by recent consensus guidelines (41); (2) Maintain regular work and rest and ensure 7–9 h of quality sleep every night; (3) Pay attention to a balanced diet and appropriately increase the intake of high-quality protein and vitamin D; (4) Make an appropriate physical activity plan according to individual circumstances. This study still has the following limitations: (1) The sample size is too small, which may affect the stability of statistical results; (2) It is a single-center study, and the extrapolation of the results needs to be verified; (3) Without long-term follow-up of patients, the effect of intervention measures cannot be evaluated. Though our sample size met the minimum statistical requirements based on power analysis, we acknowledge that the relatively small sample size (*n* = 65) may limit the generalizability of our findings. Future studies with larger samples are needed to validate our results. Therefore, it is suggested that a large sample and multi-center prospective cohort study should be carried out in the future to further verify the conclusion of this study.

## 5 Conclusion

This study found that lifestyle habits and physical activity levels are significantly associated with sarcopenia in older adults with T2DM. Regular exercise, regular sleep patterns, balanced diet, and high physical activity levels were associated with lower risk of sarcopenia in this population. These findings suggest that assessing lifestyle habits and physical activity levels may help identify patients at risk of sarcopenia, and promoting healthy lifestyle behaviors could potentially reduce sarcopenia risk in older adults with T2DM. Future larger-scale, prospective studies are needed to further validate these findings and explore the effectiveness of targeted interventions. Therefore, the risk of sarcopenia can be identified clinically by assessing these lifestyle habits and physical activity levels of patients, and then appropriate preventive measures, such as encouraging moderate exercise, maintaining regular routines, and eating a balanced diet, can be taken to reduce the risk of sarcopenia.

## Data Availability

The raw data supporting the conclusions of this article will be made available by the authors, without undue reservation.
